# A New Class of Uracil–DNA Glycosylase Inhibitors Active against Human and Vaccinia Virus Enzyme

**DOI:** 10.3390/molecules26216668

**Published:** 2021-11-03

**Authors:** Inga R. Grin, Grigory V. Mechetin, Rustem D. Kasymov, Evgeniia A. Diatlova, Anna V. Yudkina, Sergei N. Shchelkunov, Irina P. Gileva, Alexandra A. Denisova, Grigoriy A. Stepanov, Ghermes G. Chilov, Dmitry O. Zharkov

**Affiliations:** 1 SB RAS Institute of Chemical Biology and Fundamental Medicine, 8 Lavrentieva Avenue, Novosibirsk 630090, Russia; grin@niboch.nsc.ru (I.R.G.); mechetin@niboch.nsc.ru (G.V.M.); rustem.kasymov@topdatascience.com (R.D.K.); e.diatlova@g.nsu.ru (E.A.D.); ayudkina@niboch.nsc.ru (A.V.Y.); 2 Department of Natural Sciences, Novosibirsk State University, 2 Pirogova Street, Novosibirsk 630090, Russia; snshchel@rambler.ru; 3 State Research Center of Virology and Biotechnology VECTOR, Koltsovo 630559, Novosibirsk Region, Russia; gileva@vector.nsc.ru; 4 Faculty of Bioengineering and Bioinformatics, Lomonosov Moscow State University, 1 Leninskie Gory, Moscow 119991, Russia; Alexandra.Denisova@student.msu.ru; 5 National Research University Higher School of Economics, 20 Myasnitskaya Street, Moscow 101000, Russia; gastepanov@edu.hse.ru; 6 Zelinsky Institute of Organic Chemistry, Russian Academy of Sciences, 47 Leninsky Avenue, Moscow 119334, Russia; ghermes.chilov@gmail.com

**Keywords:** DNA repair, uracil–DNA glycosylase, inhibitors, virtual screening, pyrimidines

## Abstract

Uracil–DNA glycosylases are enzymes that excise uracil bases appearing in DNA as a result of cytosine deamination or accidental dUMP incorporation from the dUTP pool. The activity of Family 1 uracil–DNA glycosylase (UNG) activity limits the efficiency of antimetabolite drugs and is essential for virulence in some bacterial and viral infections. Thus, UNG is regarded as a promising target for antitumor, antiviral, antibacterial, and antiprotozoal drugs. Most UNG inhibitors presently developed are based on the uracil base linked to various substituents, yet new pharmacophores are wanted to target a wide range of UNGs. We have conducted virtual screening of a 1,027,767-ligand library and biochemically screened the best hits for the inhibitory activity against human and vaccinia virus UNG enzymes. Although even the best inhibitors had IC_50_ ≥ 100 μM, they were highly enriched in a common fragment, tetrahydro-2,4,6-trioxopyrimidinylidene (PyO3). In silico, PyO3 preferably docked into the enzyme’s active site, and in kinetic experiments, the inhibition was better consistent with the competitive mechanism. The toxicity of two best inhibitors for human cells was independent of the presence of methotrexate, which is consistent with the hypothesis that dUMP in genomic DNA is less toxic for the cell than strand breaks arising from the massive removal of uracil. We conclude that PyO3 may be a novel pharmacophore with the potential for development into UNG-targeting agents.

## 1. Introduction

Uracil–DNA *N*-glycosylases are DNA repair enzymes found in all domains of life [1,2]. They remove uracil (Ura) bases, which appear in DNA either by the spontaneous deamination of cytosines or through incorporation of dUMP from the metabolic dUTP pool [2,3]. Several classes of enzymes with this activity have been described, including the major (or Family 1) uracil–DNA glycosylase (UNG), single-strand specific monofunctional uracil DNA glycosylase (SMUG1), G/T mismatch-specific thymine DNA glycosylase (TDG), and methyl-CpG-binding domain protein 4 (MBD4). The hydrolysis of an *N*-glycosidic bond in dU nucleosides by UNG produces an abasic site (AP site) and initiates the base excision repair (BER) pathway. Then, DNA is broken 5′ to the AP site by an AP endonuclease and processed by the 2′-deoxyribo-5′-phosphate lyase activity of DNA polymerases β or λ. An undamaged nucleotide is inserted by a DNA polymerase, and the integrity of DNA is restored by a DNA ligase [4,5]. In human cells, UNG is the primary DNA glycosylase removing Ura, whereas SMUG1, TDG, and MBD4 either have specialized roles or are believed to provide a back-up [2,3,6].

Consistent with its role in replication-associated repair, UNG limits the efficiency of antimetabolite drugs that cause Ura accumulation in DNA, such as methotrexate (MTX), pemetrexed, fludarabine, and 5-fluorodeoxyuridine [7,8,9]. The up-regulation of UNG is associated with pemetrexed resistance in non-small cell lung carcinoma, while UNG knockdown sensitizes these cells to pemetrexed [10,11]. Bacterial Ung is required for the infection of macrophages by *Pseudomonas aeruginosa* and *Mycobacterium smegmatis*, apparently to cope with DNA damage by reactive nitrogen species produced by the macrophage’s oxidative burst [12]. In trypanosomes, UNG knockout results in hypersensitivity to MTX and decreased infectivity in the mouse model [13]. Finally, viral-encoded UNGs are essential for the viability and virulence of herpesviruses and poxviruses [14,15,16,17], and host-encoded UNG is important for early stages of HIV-1 replication [18,19,20]. Thus, uracil–DNA glycosylases may be regarded as promising drug targets in many conditions of neoplastic and infectious etiology.

Several attempts to develop UNG inhibitors have been reported. Most of them follow the ideas of rational drug design and build on the Ura base as a presumed pharmacophore, adding various radicals to improve the affinity or selectivity (Figure S1). For example, *N*1-substituted 6-(*p*-alkylanilino)uracils were developed into moderate-potency (IC_50_ ≈10 μM) inhibitors of UNGs from human herpes simplex virus 1 and *Plasmodium falciparum* [21,22,23,24,25]. Bipartite inhibitors, in which Ura is linked to a multiple-substituted phenyl ring, were far better in terms of potency, the best reported compound achieving submicromolar IC_50_ for the human enzyme (hUNG) [26,27,28,29]. Complexes of hUNG with several bipartite inhibitors were crystallized, revealing the expected position of Ura in the lesion-binding pocket and stabilizing interactions of the phenyl fragment in the DNA-binding groove [28,29]. Furthermore, triskelion libraries, which combine one or two Ura bases with two or one substituted phenyl rings, respectively, yielded several inhibitors of hUNG with IC_50_ ≈1 μM [30]. Of the low-molecular-weight compounds having no similarity to Ura, an aminoglycoside antibiotic gentamicin has been shown to inhibit *E. coli* UNG albeit rather weakly (IC_50_ ≈1 mM) [31,32,33]. Recently, aurintricarboxylic acid, a negatively charged compound with three conjugated aromatic rings, was shown to selectively bind hUNG in an open (i.e., not DNA-bound) conformation with submicromolar affinity [34].

Despite these efforts, the development of efficient and selective UNG inhibitors is still in want of additional pharmacophores preferably distinct from those already discovered. In this work, we used high-performance docking-based virtual screening of a chemical library to search for new UNG inhibitors. Hits identified by molecular docking were biochemically screened for the ability to inhibit the enzymatic activity of human and vaccinia virus UNGs. The mode of inhibition was determined in kinetic experiments, and the toxicity for human cells was characterized with and without antimetabolite treatment. Although the identified inhibitors had modest affinity (*K*_i_ = 140 μM at best), they all possessed a common fragment, tetrahydro-2,4,6-trioxopyrimidinylidene (PyO3), a 5-substituted barbituric acid derivative, which may be considered as a new promising pharmacophore.

## 2. Results

### 2.1. Virtual Screening

**Figure 1:**
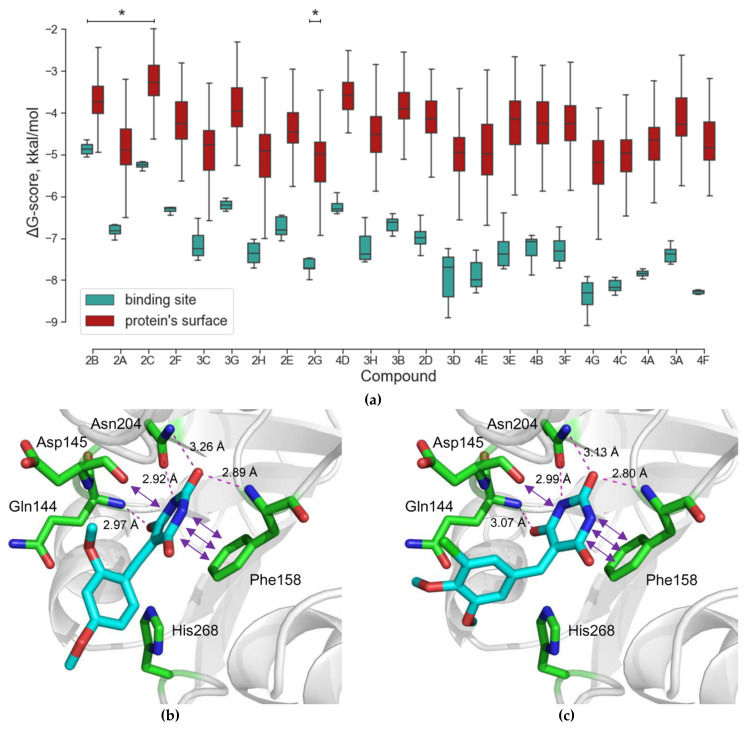
Docking of inhibitors into hUNG structure. (**a**) Illustration of the On-Top Docking procedure: the ΔG-score calculated by Lead Finder for each of the selected compounds for a set of poses inside the binding site (cyan bars) and on the protein surface (red bars); * marks the potentially inactive compounds, for which *p*_Median Test_ > 0.01 (see Table S1). (**b**) Structure of hUNG (PDB ID 3FCF [29]) with compound 4B docked into the active site. Lengths of the stabilizing hydrogen bonds are shown. Double-headed arrows indicate interactions with the π-system of the PyO3 moiety. (**c**) Same as (b) but with compound 4F docked into the hUNG active site.

The virtual library of 1,027,767 compounds was docked into the hUNG structure (PDB ID 3FCF [29]). Specific hydrogen bonds were found only in 1124 complexes. On-Top Docking was applied to 671 low-molecular-weight compounds (Figure 1a, Supplementary Data S1, Supplementary Data S2), and 378 potentially active ligands were selected. To evaluate the experimental activity, 19 potentially active ligands were selected, and four potentially inactive ligands were included as controls (Figure S2, Table S1). The personnel involved in all aspects of the biochemical screening (planning, conducting, and data analysis) was kept blinded with respect to the presence of the potentially inactive ligands; the compounds were randomly encoded in an alphanumerical manner (e.g., 2A, 4B, etc.).

The structures of the complex of hUNG with compounds 4B and 4F (Figure 1b,c) provide characteristic examples of the dense network of hydrogen bonds formed by the low-molecular-weight compound in the enzyme’s active site. Additional stabilization of the structure is provided by π–π stacking with Phe158 and σ–π dative interaction with Asp145, which are not directly accounted for in the Lead Finder calculations.

### 2.2. Biochemical Screening

**Figure 2:**
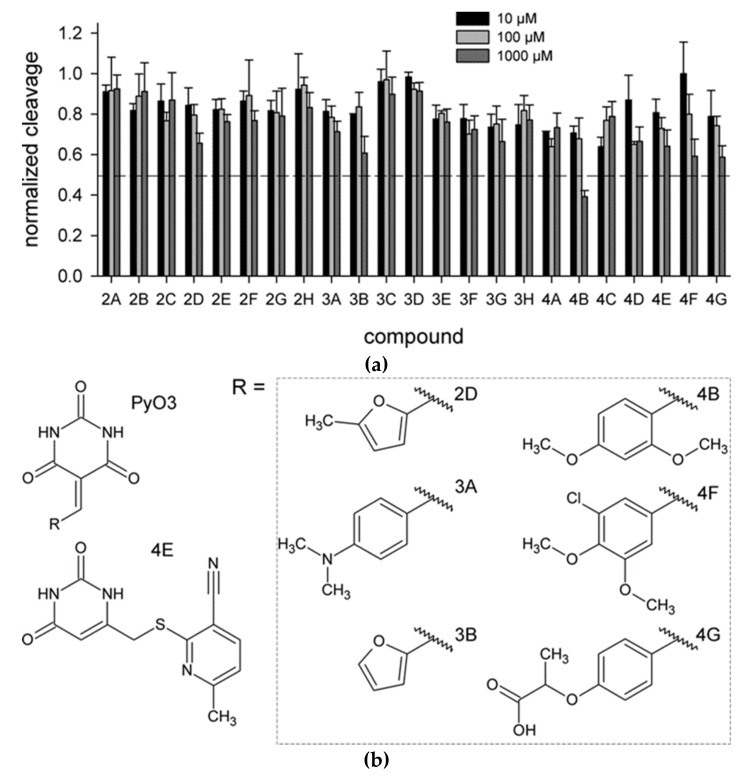
Biochemical screening of the inhibitors identified by molecular docking. (**a**) hUNG activity in the presence of the inhibitors at 10 μM (black bars), 100 μM (light gray bars), or 1000 μM (dark gray bars). The activity is normalized for that in the absence of the inhibitor. Means and SD of three independent experiments are shown; (**b**) Structures of the compounds 2D, 3A, 3B, 4B, 4E, 4F, and 4G.

As hUNG excises Ura from single- and double-stranded DNA with similar efficiency [6,35], we have used the cleavage of a 20-mer single-stranded DNA oligonucleotide containing a dU residue in position 8 (Section 4.1) to assay hUNG activity in the presence of inhibitors. Twenty-three identified compounds were tested at 10, 100, and 1000 μM (Figure 2a). Six of the prospective hUNG inhibitors (2D, 3A, 4B, 4E, 4F, and 4G) demonstrated progressively lower substrate cleavage with the increasing inhibitor concentration. Notably, all but one of these compounds contained a common fragment, PyO3, and they were different only in substituents at the aromatic ring (Figure 2b). However, even the best inhibitor, 4B, had IC_50_ between 100 and 1000 μM under the conditions used for screening. None of the potentially inactive ligands inhibited hUNG in the biochemical screening.

### 2.3. Character of UNG Inhibition

**Figure 3:**
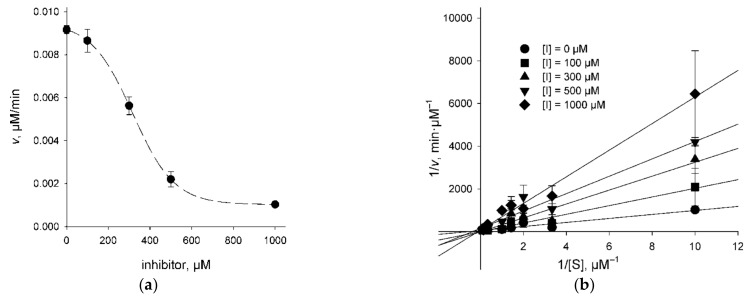
The mode of UNG inhibition. (**a**) Cleavage of the substrate (1 μM) by hUNG at different concentrations of compound 4B; (**b**) cleavage by hUNG at different concentrations of the substrate and compound 4B plotted in double reciprocal coordinates.

To get an idea of whether the UNG inhibitors indeed bind at the lesion recognition site of the enzyme, we have determined the mode of inhibition for 3A (which showed a weak dose response), 4B, 4F, and 4G (all showing a more pronounced dose response) using the classic steady-state kinetic analysis. *K*_M_ and *V*_max_ parameters of the Michaelis–Menten scheme were determined at different inhibitor concentrations, and the results were analyzed by fitting to competitive, uncompetitive, and mixed inhibition models (Figure 3, Table 1). Akaike’s information criterion was used to compare the quality of fit under the models [36]. The mixed inhibition model in all cases either did not converge upon numerical non-linear fitting or produced *K*_i_ estimates with errors exceeding the mean. Inhibitors 4B and 4G were clearly better described by the competitive inhibition model (Table 1). For 3A and 4F, the uncompetitive model was marginally better. The values for *K*_M_ and *V*_max_ determined independently in experiments with different inhibitors under different models coincided quite well (*K*_M_ = 1.1–6.4 μM, average 3.2 ± 1.3 μM; *V*_max_ = 0.019–0.075 μM/min, average 0.040 ± 0.007 μM/min, corresponding to *k*_cat_ = 1990 ± 360 min^−1^ at 20 pM hUNG), confirming the validity of the fits. The kinetic values reported for ssDNA cleavage by hUNG in the literature vary widely (0.22–13.8 μM for *K*_M_, 647–1060 min^−1^ for *k*_cat_) [6,37,38] yet generally agree with our data.

### 2.4. UNG Inhibitors Toxicity Is Independent of Methotrexate

**Figure 4:**
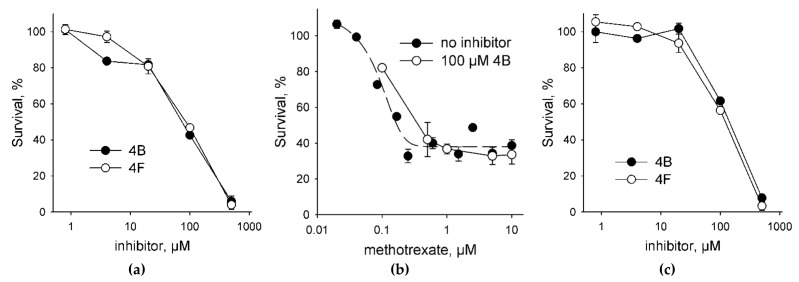
Cytotoxicity of UNG inhibitors. (**a**) Survival of HEK 293FT cells in the presence of the UNG inhibitors 4B and 4F (0.8–500 μM); (**b**) survival of HEK 293FT cells in the presence of MTX alone (0.02–10 μM) or 100 μM 4B plus MTX (0.1–10 μM); (**c**) survival of HEK 293FT cells in the presence of the UNG inhibitors 4B and 4F (0.8–500 μM) plus 0.1 μM MTX. In Panel C, the survival in the presence of 0.1 μM MTX alone is taken for 100%. Mean ± s. e. m. is shown (*n* = 3).

To assess the toxicity of UNG inhibitors for human cells, we have treated HEK293 cell line with the compounds 4B and 4F as the best inhibitors (Table 1). Both compounds demonstrated a typical dose–response and a moderate toxicity (Figure 4a); the effective concentrations were 69 ± 1 μM for 4B and 77 ± 1 μM for 4F. Since Ura preferentially accumulates in DNA from a dUTP pool, we have next tested the toxicity of these UNG inhibitors in the presence of MTX, which is an antimetabolite drug that inhibits dihydrofolate reductase, reduces the availability of tetrahydrofolate, and thus suppresses the conversion of dUTP to dTTP. At 0.1 μM MTX without the inhibitors, we have observed ≈20–25% reduction in the cell viability (Figure 4b), which is in good agreement with the EC_50_ values for 947 human cell lines for this drug (median EC_50_ = 0.595 μM) collected by the Genomics of Drug Sensitivity in Cancer Project [39]. In the presence of 0.1 μM MTX, both 4B and 4F showed a 1.5–2-fold increase in the effective concentration (Figure 4c; EC_50_ = 140 ± 10 μM for 4B and 110 ± 10 μM for 4F). Moreover, an increase in the MTX concentration in the presence of a fixed amount of an inhibitor did not produce additional cell killing compared to MTX alone (Figure 4b). Although we did not evaluate the accumulation of Ura in the genomic DNA of the treated cells, this behavior is consistent with the model in which the incorporation of dUMP is less toxic for the cell than strand breaks arising from the massive removal of Ura from the genome [3,40].

### 2.5. Inhibition of Vaccinia Virus UNG

**Figure 5:**
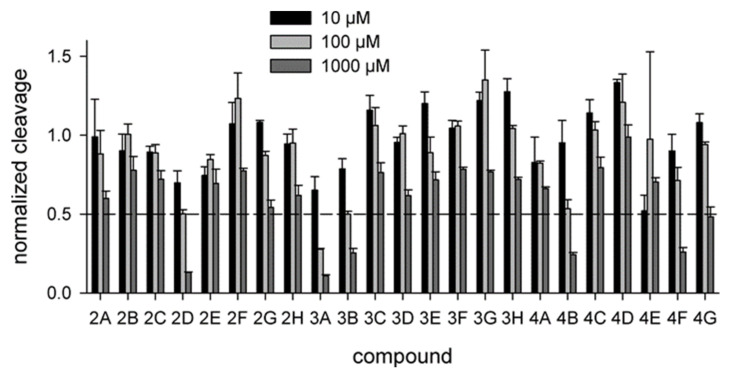
Biochemical screening of the inhibitors against vvUNG. The inhibitor concentration was 10 μM (black bars), 100 μM (light gray bars), or 1000 μM (dark gray bars). The activity is normalized for that in the absence of the inhibitor. Means and SD of three independent experiments are shown.

**Figure 6:**
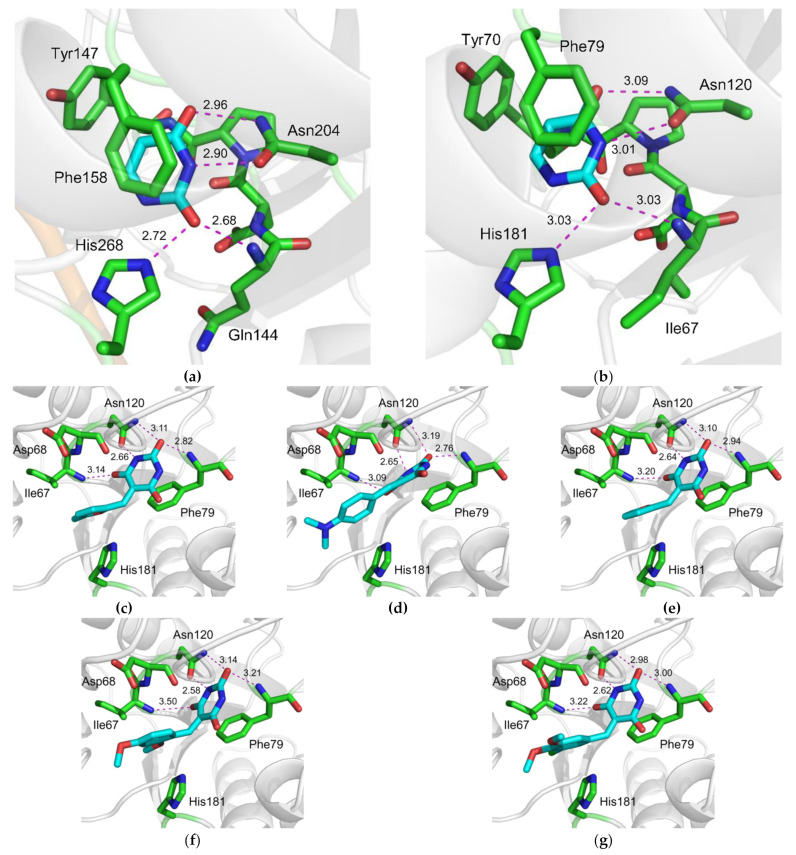
Structures of the uracil-binding pockets of (**a**) hUNG (Protein Data Bank ID 1SSP [44]) and (**b**) vvUNG (4YIG [47]) with the excised Ura base. Both structures also contain the abasic DNA, which is not shown for clarity. (**c**–**g**) Structure of vvUNG uracil-binding pocket with docked compounds 2D (**c**), 3A (**d**), 3B (**e**), 4B (**f**), and 4F (**g**). Dashes indicate hydrogen bonds; their length in Å is shown. The carbon atoms of the excised uracil base (**a**–**b**) and the inhibitors (**c**–**g**) are colored cyan; protein carbons are green.

All uracil–DNA glycosylases share a common fold and possess a highly conserved active site. To see whether the compounds identified by in silico screening against hUNG could also bind and inhibit the enzyme from other species, we have tested their ability to interfere with the activity of vaccinia virus UNG (vvUNG, also known as D4 protein). vvUNG is both an efficient Ura removal enzyme and a processivity subunit of the viral replicative machinery [41,42]. The inhibitors were assayed under the same conditions as for hUNG. Surprisingly, vvUNG was inhibited even to a higher degree than hUNG: thirteen compounds showed some kind of a dose response, and four of them (2D, 3A, 3B, 4B) suppressed the activity of vvUNG by ≈50% at 100 μM (Figure 5). Again, all the best inhibitors contained PyO3 as a common fragment (Figure 2b). This is not surprising, given that the uracil-binding sites of hUNG and vvUNG are quite similar (Figure 6a,b). We have performed docking of the apparently most potent vvUNG inhibitors (2D, 3A, 3B, 4B, and 4F) to vvUNG. In all cases, the inhibitor molecule well fit the uracil-binding pocket, forming interactions with the residues (Ile67, Asp68, Phe79, and Asn120) corresponding to the inhibitor-binding residues in the human enzyme (Figure 6c–g).

## 3. Discussion

Most known UNG inhibitors contain an Ura moiety in their structure and are presumed to act through competition with damaged DNA for the Ura-binding pocket of the enzyme. UNG owes its exquisite specificity to the pocket’s shape being highly complementary to the structure of Ura (Figure 6a,b). In hUNG, the Ura base is extruded from the DNA helix and squeezed between the plane of Phe158 and the ^144^QNP^146^ loop [43,44,45]. Tyr147 phenyl ring occupies the space near C5 of Ura, allowing the enzyme to reject thymine, which has a methyl group at this position. At the same time, the Asn204 side chain forms hydrogen bonds to N3 and O^4^ of Ura, thus discriminating against cytosine [43,44]. His268 and the main chain amide of Gln144 complete the set of interactions with Ura, donating non-discriminating but catalytically important hydrogen bonds to O^2^. All these interactions are conserved in the structures of UNG bound to a series of bipartite inhibitors [29]. The pocket opens in the direction of N1 and C6 of Ura, and the C6 position carries substituent radicals in all Ura-derived UNG inhibitors (reviewed in [46]).

We have found that 5-substituted PyO3 derivatives inhibit UNG. Our screened panel included nine out of 23 compounds containing a Ura moiety and six out of 23 compounds containing a PyO3 moiety. PyO3 was significantly overrepresented among the fragments found in the active compounds for both hUNG and vvUNG (*p* = 0.001 and 0.048, respectively, by Fisher’s exact test; *p* = 0.0017 for vvUNG inhibitors with IC_50_ ≈100 μM). Ura was neither over- nor underrepresented among the active compounds. Structurally, PyO3 resembles barbituric acid, but due to the lack of hydrogens at C5, it is not acidic. The substitution at C5 favors binding in an orientation resembling binding of a non-hydrolyzable pseudouridine analog in the active site of UNG [45]. In this structure, the Tyr147 ring is positioned next to a nitrogen atom instead of C5, without apparent steric collisions. A dense network of hydrogen bonds involving Gln144, Phe158, and Asn204 contributes to the stabilization of the complex. As with the everted uracil, additional stabilizing π–π stacking with Phe158 and a σ–π dative interaction with Asp145 are observed in the docked structure (Figure 1, Supplementary Data S3). In vvUNG, the inhibitors contacted a set of pocket residues homologous to the hUNG inhibitor-interaction residues (Figure 6c–g), supporting a common recognition mechanism.

The two best inhibitors, 4B and 4F, demonstrated IC_50_ and *K*_i_ values in the submillimolar range for both the competitive and uncompetitive model. A comparison with the UNG affinities for Ura and Ura-derived inhibitors [21,27] suggests that these values are consistent with the contribution from the heterocyclic base without significant additional stabilization. Extensive modification of the linker and the linked aromatic moiety was required to achieve submicromolar to micromolar IC_50_ with Ura-derived inhibitors [27,28,29,30]. Importantly, the structures of UNG bound to a series of bipartite inhibitors [28,29] show that the best inhibitors (IC_50_ < 1.6–40 μM) stack a benzoic acid moiety against the catalytic His268, while the worse ones (IC_50_ > 100 μM) did not have apparent additional stabilizing interactions. In our case, the two best inhibitors (4B and 4F) interact with His268 via a bridging water molecule. The resorcinol moieties in both molecules are close to His268 but form no stabilizing interactions. In addition, one of the hydroxyalkyl groups of 4F accepts a hydrogen bond from the side chain of Ser169. Therefore, an increase in the strength of the inhibitor is likely associated with the steric inaccessibility of His268 and additional hydrogen bonds between the ligand and the active site of the protein (Figure 1). Interestingly, compounds 4B and 4F, despite their structural similarity, might have different kinetic mechanisms of inhibition: while 4B clearly demonstrated competitive behavior, 4F was somewhat better described by an uncompetitive model (Table 1). This is reminiscent of the behavior of bipartite UNG inhibitors, which, depending on the nature of the linker and the stabilizing aromatic moiety, may show competitive, partial uncompetitive, and partial mixed-type inhibition mode [27]. The reasons for such differences are not completely clear. The structures of inhibitors- and DNA-bound UNG [28,29,43,44,45,48] suggest that all inhibitors should be at least partially competitive, since their binding is incompatible with the simultaneous productive binding of damaged DNA. One interesting possibility is that the inhibitors might interact with the “early recognition” UNG–DNA complex [48], in which the uracil-binding pocket remains accessible, and the bound DNA with a partially everted normal nucleobase provides additional possibilities for stabilizing contacts. UNG is naturally not allosterically regulated, making it less likely that the enzyme possesses functional binding sites for low-molecular-weight ligands other than the active site and the early recognition site.

In the cell culture, the inhibitors showed moderate toxicity that was apparently not potentiated by MTX, a dihydrofolate reductase inhibitor that elevates intracellular dUTP and promotes the accumulation of Ura in DNA [49,50]. At present, it is impossible to say whether the observed toxicity of 4B and 4F is caused by the consequences of hUNG inhibition such as the accumulation of Ura in the genomic DNA. Although in several cancer cell lines UNG was reported to mediate the resistance to antimetabolites [7,8,9,10,11,51,52], the general correlation between the cellular dUTP pool size, the efficiency of genomic Ura repair, and cell viability is not straightforward. Whereas Ura repair is required to maintain the genome integrity under normal damage load, it might be detrimental when the damage is excessive due to the conversion of Ura to more toxic strand breaks. In *E. coli*, *dut* mutations that inactivate deoxyuridine triphosphatase and increase the dUTP pool are synthetically lethal with UNG overexpression, whereas *ung* mutation partially rescues growth defects and chromosome fragmentation in the *dut* background [53,54]. Fetal neocortical and hippocampal neurons but not embryonic fibroblasts from *ung^−/−^* knockout mice show enhanced sensitivity to MTX [55,56]. No increase in the sensitivity to raltitrexed, an MTX analog, is observed in chicken *ung^−/−^* DT40 lymphoma cells [57]. Importantly, in the HEK293 cell line, which was used in this work, the expression of Ugi, a protein inhibitor of UNG, does not change the raltitrexed sensitivity despite a ≈4-fold increase in the genomic Ura content upon the drug treatment [58]. Further studies are required to assess the levels of genomic Ura in the presence of PyO3-based UNG inhibitors, as well as the cellular response to the inhibitors combined with pyrimidine antimetabolites that can be incorporated into DNA, such as 5-fluorouracil and 5-fluoro-2′-deoxyuridine, which are known to be repaired by UNG [9,51,52].

VvUNG binds viral DNA polymerase and A20 protein to form the viral replicative complex [41,42,59]. Interestingly, hUNG also interacts with the replicative complex, presumably through proliferating cell nuclear antigen, and it is cell-cycle regulated through phosphorylation and proteolysis, peaking in the S phase [60,61]. So far, the only small-molecule ligands directed against vvUNG targeted the interface between vvUNG and A20, which is another subunit of the viral replication complex [62,63]. However, this interface does not overlap with the active site or the DNA-binding cleft of vvUNG [47,59]. The catalytic activity of vvUNG is disposable for virus replication but essential for its virulence [15,17,64], so the inhibition of vvUNG can be regarded as a promising antiviral strategy. It remains to be seen whether the inhibition of vvUNG by PyO3 derivatives affects its ability to serve as a processivity subunit.

Overall, our study has revealed a possible new pharmacophore, tetrahydro-2,4,6-trioxopyrimidinylidene, which can bind to and inhibit human and viral uracil–DNA glycosylase. Linking the PyO3 moiety to additional groups designed to stabilize the interactions in the DNA-binding groove may further improve its binding affinity and selectivity toward UNGs from different species.

## 4. Materials and Methods

### 4.1. Enzymes and Oligonucleotides

Restriction endonucleases were purchased from New England Biolabs (Ipswich, MA, USA), and phage T4 DNA ligase were purchased from Thermo Fisher Scientific (Waltham, MA, USA). The substrate oligodeoxyribonucleotide 5′-GGACTTCUCTCCTTTCCAGA-3′ was synthesized in-house from commercially available phosphoramidites (Glen Research, Sterling, VA, USA). The substrate was ^32^P-labeled using γ[^32^P]ATP (SB RAS ICBFM Laboratory of Biotechnology, Novosibirsk, Russia) and phage T4 polynucleotide kinase (Biosan, Novosibirsk, Russia) according to the manufacturer’s protocol and purified by reverse-phase chromatography on C_18_ NenSorb sorbent (DuPont, Wilmington, DE, USA). VvUNG cloning and purification will be described elsewhere (Diatlova et al., paper in preparation) but generally followed the procedure from [65].

The sequence of the truncated catalytically active human uracil–DNA glycosylase (hUNG ΔN84) was amplified from the pTRC99A-UNGΔ84 [35] (a kind gift from Dr. Hans Krokan, University of Trondheim, Norway) and re-cloned into the pET-15b plasmid at *Nde*I–*Xho*I sites. The resulting construct encodes hUNG ΔN84 with an N-terminal His_6_-tag and a short linker containing a thrombin cleavage site. To purify the protein, *E. coli* BL21(DE3) cells transformed with this plasmid was grown in 1 l of the LB broth supplemented with 100 μg/mL ampicillin at 37 °C to A_595_ = 0.6 and induced with 1 mM isopropyl β-d-1-thiogalactopyranoside for 3 h. The cells were collected by centrifugation at 4 °C, resuspended in 20 mL of the lysis buffer (10 mM Tris–HCl pH 8.0, 1 mM EDTA, 500 mM NaCl, 1 mM phenylmethylsulfonyl fluoride), and lysed by sonication. Cell debris was separated by centrifugation at 15,000× *g* for 30 min at 4 °C; then, the supernatant was treated with ammonium sulfate (60% saturation), and centrifuged again. The protein pellet was dissolved in 20 mL of buffer A (20 mM Tris-HCl pH 7.5; 500 mM NaCl; 20 mM imidazole), the solution was filtered through a 0.45 µm Millex-HV filter (Merck Millipore, Burlington, MA, USA) and loaded onto a HiTrap Chelating column (GE Healthcare, Chicago, IL, USA) charged with Ni^2+^ ions and equilibrated in the same buffer. The bound protein was eluted using a gradient of 20–500 mM imidazole. Fractions containing the target protein were identified by SDS-PAGE with Coomassie Blue staining. The fractions were diluted with 20 mM Tris–HCl (pH 7.5) to ≈50 mM NaCl, loaded onto a 1 mL HiTrap Heparin Sepharose column (GE Healthcare), and the bound protein was eluted with a 25 mL gradient of 0–1000 mM in 20 mM Tris–HCl (pH 7.5). The fractions containing the target protein were dialyzed overnight against the buffer containing 20 mM sodium phosphate (pH 7.5), 400 mM NaCl, 1 mM EDTA, 1 mM dithiothreitol, and 50% glycerol, and stored at –20 °C.

### 4.2. Virtual Screening

A set of 1,027,767 ligands comprising the STK library of Vitas-M Laboratory (Vitas-M Laboratory, Ltd., Hong Kong, China) was used in virtual screening. For the molecular modeling purposes, the 3D coordinates of inhibitors were generated with RDKit (www.rdkit.org, rdkit release 5 March 2021, assessed on 31 March 2021). For each molecule, the p*K*_a_ values of all ionizable groups were calculated, and then, the ionization states were set to those corresponding to pH 7.0. The full-atom models of hUNG in complex with a Ura-based inhibitor (PDB ID 3FCF [29]) or of vvUNG (4YIG [47]) were prepared by adding polar hydrogen atoms and assigning the ionization states of the amino acids by the Build Model module that is part of the Lead Finder software package [66,67]. Each model was validated by docking its cognate ligand (extracted from the corresponding PDB structure) and measuring the root mean square deviation (RMSD) of the docked ligand pose from its crystallographic position. Virtual screening calculations were performed with Lead Finder software v1.1.16 [68] using default configuration parameters. Only the top-ranked poses were used for further analysis. For each top-scoring ligand pose obtained in virtual screening, the formation of a particular set of specific protein–ligand interactions (H-bonds or coordination to metal ion) was monitored. Only protein–ligand complexes with four particular hydrogen bonds (Figure 1) were kept for further evaluation according to the previously described protocol [69].

Our custom developed technique called On-Top Docking (to be published) was used for ranging the compounds’ binding affinities calculated by Lead Finder. Briefly, On-Top Docking explicitly estimates the energy of ligand interaction with the protein surface outside the ligand binding site and uses it as a baseline for assessing the actual binding energy inside the active site (Figure S1). The ligand is considered potentially active if its binding affinity inside the active site exceeds its binding affinity in an arbitrary site on the protein surface, as evaluated by the Median Test with 1% significance point. This procedure is designed to reduce the rate of false negative predictions, which is the major source of errors in virtual screening.

Molecular structure images were prepared using PyMol (Schrödinger, New York, NY, USA).

### 4.3. Uracil–DNA Glycosylase Assay

The identified compounds were purchased from Vitas-M Laboratory (Vitas-M Laboratory, Ltd., Hong Kong, China). The reaction mixture (10 μL) included 25 mM sodium phosphate (pH 7.5), 1 mM dithiothreitol, 1 mM Na-EDTA, 50 nM ^32^P-labeled substrate, 20 pM enzyme, and the inhibitors at 10, 100, and 1000 μM. The reaction was carried out for 10 min at 37 °C, terminated by adding NaOH to 100 mM and heating for 2 min at 95 °C, and neutralized by an equimolar amount of HCl. The mixture was supplemented with 5 μL of formamide dye (80% formamide, 20 mM Na-EDTA, 0.1% xylene cyanol, 0.1% bromophenol blue), and the reaction products were separated by gel electrophoresis in 20% polyacrylamide/7.2 M urea. The gels were visualized and quantified by phosphorimaging using the Typhoon FLA 9500 system (GE Healthcare, Chicago, IL, USA).

### 4.4. Inhibition Mechanism Determination

The reactions were performed as described above except that the substrate concentrations were 0.1, 0.3, 0.5, 0.7, 1, 3, 5, 7, and 10 μM, and the inhibitors concentrations were 0, 100, 300, 500, and 1000 μM. The time of incubation was adjusted so that the substrate cleavage did not exceed 15%. To determine the type of inhibition and calculate *K*_i_, the data were fit to the competitive, uncompetitive, and mixed inhibition models using an R script [70].

### 4.5. Cell Toxicity Assay

Human embryonic kidney cells (HEK 293FT) were from Thermo Fisher Scientific (Waltham, MA, USA; Cat. #R70007). The laboratory stock was regularly checked for the absence of mycoplasma contamination by PCR. Cells were grown in T25 cm^2^ flasks in the complete medium (Dulbecco’s modified Eagle’s medium supplemented with 10% fetal bovine serum, 1 mM sodium pyruvate, 2 mM L-glutamine, and 1% penicillin–streptomycin–amphotericin B) as adherent monolayer cultures. The cultures were maintained at 37 °C in humidified atmosphere with 5% CO_2_. Cytotoxicity was determined by the colorimetric MTT (3-(4,5-dimethyl-2-thiazolyl)-2,5-diphenyl-2*H*-tetrazolium bromide) assay [71]. The cells were harvested by trypsinization, seeded in the complete medium (100 μL per well) into 96-well plates at the density of 1.4×10^4^ cells per well, and incubated for 24 h to settle and resume proliferation. MTX was dissolved in 1 M NaOH to 22 mM. The test compounds 2B, 4B, and 4F were dissolved in water, appropriately diluted in the complete medium containing 0.1 μM MTX, and immediately added to the cells (100 μL per well; the final concentration of NaOH from MTX stock solution was 9.1 μM, well below the medium’s buffering capacity). The control cells were treated with 0.1 μM MTX without inhibitors. After exposure for 72 h, 22 μL of MTT reagent was added to each well and incubated for 3 h at 37 °C. The supernatant was removed, and 100 μL of isopropanol was added. After 15 min at room temperature to solubilize the formed formazan, A_570_ and A_620_ were measured with a Multiskan EX microplate reader (Thermo Fisher Scientific, Waltham, MA, USA). Three independent experiments were performed with triplicates for each concentration.

## Figures and Tables

**Table 1:** Kinetic constants for substrate cleavage by hUNG in the presence of various inhibitors.

Inhibitor	Model	*θ*_rel_^a^	*V*_max_, μM/min	*K*_M_, μM	*K*_i_, μM
3A	competitive	0.655	0.029 ± 0.003	1.6 ± 0.5	530 ± 270
	uncompetitive	best model	0.034 ± 0.004	2.6 ± 0.7	1300 ± 500
4B	competitive	best model	0.019 ± 0.001	1.1 ± 0.3	140 ± 40
	uncompetitive	2.53 × 10^−5^	0.024 ± 0.003	2.9 ± 0.7	550 ± 140
4F	competitive	0.8	0.053 ± 0.014	3.1 ± 2.2	200 ± 130
	uncompetitive	best model	0.075 ± 0.028	6.4 ± 4.5	250 ± 140
4G	competitive	best model	0.042 ± 0.002	3.7 ± 0.6	3300 ± 2300
	uncompetitive	0.312	0.043 ± 0.003	4.2 ± 0.7	>10,000

^a^ Akaike’s relative likelihood that the given model describes the inhibition data better than the competing model. The best model’s likelihood is taken for 1.

## Data Availability

All data are contained in the paper and Supplementary Materials.

## Supplementary Materials

The following are available online. Figure S1: Examples of known UNG inhibitors, Figure S2: Distribution of compounds by ΔG-score calculated by Lead Finder. Coloring: red text—*potentially inactive* inhibitors, black—*potentially active* inhibitors. If the assessment of binding significantly different (Median Test with significance point 1%) exceeded the assessment of binding in an arbitrary site on the protein surface, the ligand was considered *potentially active*; other ligands were considered *potentially inactive*, Table S1: *p*-value of Median Test for the 10 best ΔG by Lead Finder score for docking in active site and in an arbitrary center, Data S1: *low_mw.sdf* file containing 671 compounds with specific hydrogen bonds in complex with hUNG and molecular weight less than 350, Data S2: *hUNG_671.zip* archive containing the results of On-Top Docking for 671 compounds, Data S3: *4B_hUNG.pdb* file containing crystal structure of hUNG (PDB ID: 3FCF) in complex with the docked 4B.

## Sample Availability

Samples of the compounds 2D, 3A, 3B, 4B, 4F, 4G are available from the authors.
