# Supplementary material for: A New Class of Uracil–DNA Glycosylase Inhibitors Active against Human and Vaccinia Virus Enzyme
Source: Molecules. 2021 Nov 3;26(21):6668. doi: 10.3390/molecules26216668 (PMC8587785; doi:10.3390/molecules26216668)
Supplement: Supplementary file 1 [file molecules-26-06668-s001.zip › Figure S2.pdf]

Supporting Information for

Grin *et al.*, A new class of uracil–DNA glycosylase inhibitors active against human and vaccinia virus enzyme

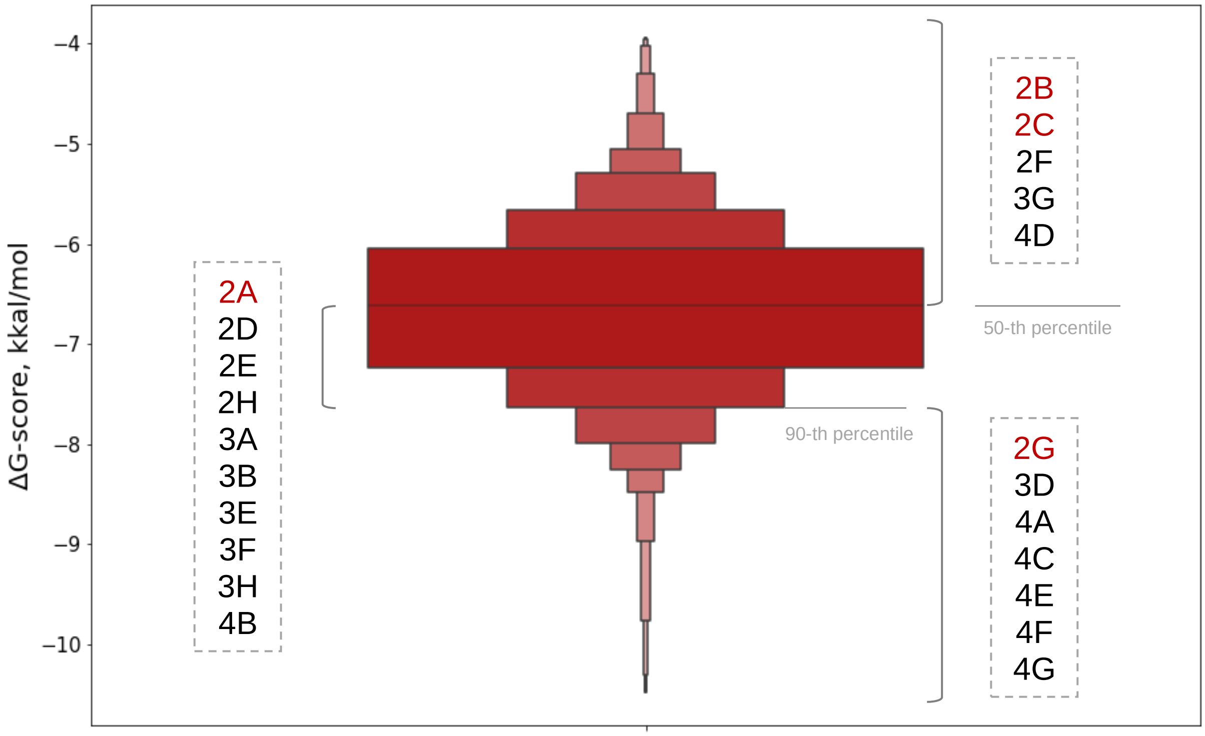

Fig. S2. Distribution of compounds by ΔG-score calculated by Lead Finder. Coloring: red text – *potentially inactive* inhibitors, black – *potentially active* inhibitors. If the assessment of binding significantly different (Median Test with significance point 1%) exceeded the assessment of binding in an arbitrary site on the protein surface, the ligand was considered as *potentially active*, other ligands were considered ad *potentially inactive*.
