# Supplementary material for: A New Class of Uracil–DNA Glycosylase Inhibitors Active against Human and Vaccinia Virus Enzyme
Source: Molecules. 2021 Nov 3;26(21):6668. doi: 10.3390/molecules26216668 (PMC8587785; doi:10.3390/molecules26216668)
Supplement: Supplementary file 1 [file molecules-26-06668-s001.zip › Table S1.pdf]

Supporting Information for

Grin *et al.*, A new class of uracil–DNA glycosylase inhibitors active against human and vaccinia virus enzyme

Compound	Median Test (*p*-value)	Compound	Median Test(*p*-value)
**2A**	0,025	**3E**	$5{,}7 \times 10^{-5}$
**2B**	1.00	**3F**	$5{,}7 \times 10^{-5}$
**2C**	0,66	**3G**	$5{,}7 \times 10^{-5}$
**2D**	$5{,}7 \times 10^{-5}$	**3H**	$5{,}7 \times 10^{-5}$
**2E**	$5{,}7 \times 10^{-5}$	**4A**	$5{,}7 \times 10^{-5}$
**2F**	$5{,}7 \times 10^{-5}$	**4B**	$5{,}7 \times 10^{-5}$
**2G**	0,025	**4C**	$5{,}7 \times 10^{-5}$
**2H**	$5{,}7 \times 10^{-5}$	**4D**	$5{,}7 \times 10^{-5}$
**3A**	$5{,}7 \times 10^{-5}$	**4E**	0,0017
**3B**	$5{,}7 \times 10^{-5}$	**4F**	$5{,}7 \times 10^{-5}$
**3C**	0,0017	**4G**	$5{,}7 \times 10^{-5}$
**3D**	$5{,}7 \times 10^{-5}$		

Table S1. p-value of Median Test for the 10 best ΔG by Lead Finder score for docking in active site and in an arbitrary center.
