# Supplementary material for: A New Class of Uracil–DNA Glycosylase Inhibitors Active against Human and Vaccinia Virus Enzyme
Source: Molecules. 2021 Nov 3;26(21):6668. doi: 10.3390/molecules26216668 (PMC8587785; doi:10.3390/molecules26216668)
Supplement: Supplementary file 1 [file molecules-26-06668-s001.zip › Figure S1.pdf]

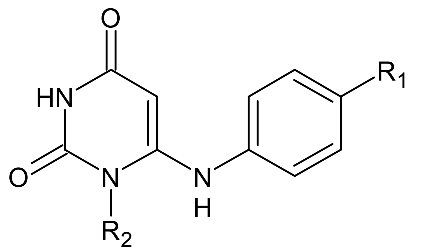

6-(*p*-alkylanilino)uracils

$R_1$ = *n*-octyl

$R_2$ = H; 1-methoxyethyl

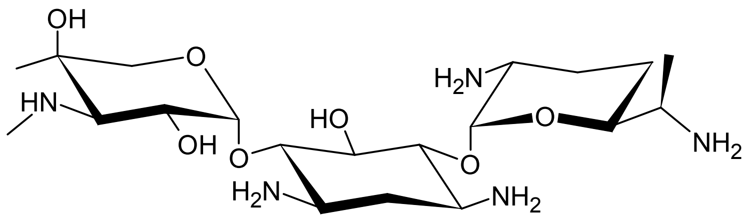

Gentamicin

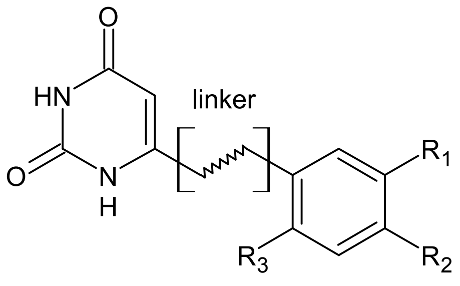

Bipartite inhibitors

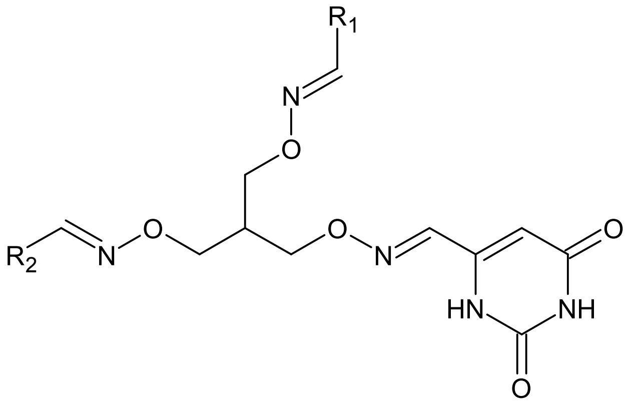

Triskelion inhibitors

linker

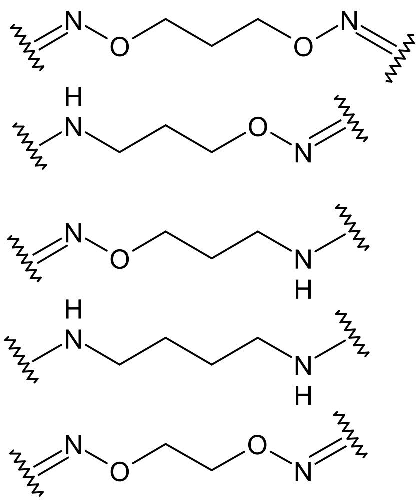

$R_1$ = H; OH; COOH

$R_2$ = H; COOH

$R_3$ = H; OH; F; Cl; Br; $NO_2$

$R_1$, $R_2$ =

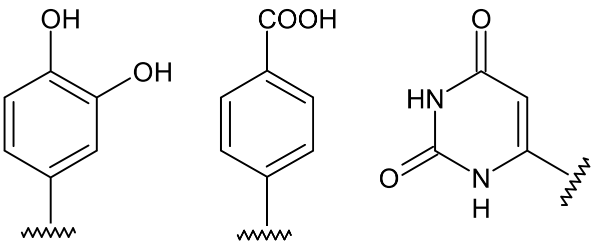

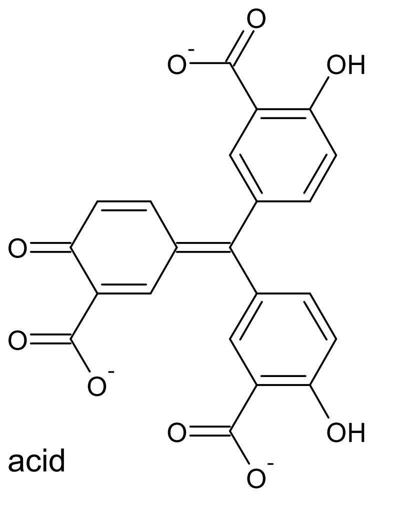

Aurintricarboxylic acid

Figure S1
